# Determination of optimal ultrasound planes for the initialisation of image registration during endoscopic ultrasound-guided procedures

**DOI:** 10.1007/s11548-018-1762-2

**Published:** 2018-04-16

**Authors:** Ester Bonmati, Yipeng Hu, Eli Gibson, Laura Uribarri, Geri Keane, Kurinchi Gurusami, Brian Davidson, Stephen P. Pereira, Matthew J. Clarkson, Dean C. Barratt

**Affiliations:** 1 UCL Centre for Medical Image Computing, University College London, London, UK; 2 Wellcome/EPSRC Centre for Interventional and Surgical Science, University College London, London, UK; 3 Institute for Liver and Digestive Health, University College London, London, UK; 4 Division of Surgery and Interventional Science, University College London, London, UK

**Keywords:** EUS, Planning, Image registration, Computer-assisted interventions, Pancreatic cancer

## Abstract

**Purpose:**

Navigation of endoscopic ultrasound (EUS)-guided procedures of the upper gastrointestinal (GI) system can be technically challenging due to the small fields-of-view of ultrasound and optical devices, as well as the anatomical variability and limited number of orienting landmarks during navigation. Co-registration of an EUS device and a pre-procedure 3D image can enhance the ability to navigate. However, the fidelity of this contextual information depends on the accuracy of registration. The purpose of this study was to develop and test the feasibility of a simulation-based planning method for pre-selecting patient-specific EUS-visible anatomical landmark locations to maximise the accuracy and robustness of a feature-based multimodality registration method.

**Methods:**

A registration approach was adopted in which landmarks are registered to anatomical structures segmented from the pre-procedure volume. The predicted target registration errors (TREs) of EUS-CT registration were estimated using simulated visible anatomical landmarks and a Monte Carlo simulation of landmark localisation error. The optimal planes were selected based on the 90th percentile of TREs, which provide a robust and more accurate EUS-CT registration initialisation. The method was evaluated by comparing the accuracy and robustness of registrations initialised using optimised planes versus non-optimised planes using manually segmented CT images and simulated ($n=9$) or retrospective clinical ($n=1$) EUS landmarks.

**Results:**

The results show a lower 90th percentile TRE when registration is initialised using the optimised planes compared with a non-optimised initialisation approach (*p* value $< 0.01$).

**Conclusions:**

The proposed simulation-based method to find optimised EUS planes and landmarks for EUS-guided procedures may have the potential to improve registration accuracy. Further work will investigate applying the technique in a clinical setting.

**Electronic supplementary material:**

The online version of this article (10.1007/s11548-018-1762-2) contains supplementary material, which is available to authorized users.

## Introduction

Endoscopic ultrasound (EUS) is a minimally invasive technique to guide interventional procedures to evaluate and treat pancreatobiliary disorders, including pancreatic cancer [1]. EUS provides a safe and effective means of identifying cancer and can be combined with fine needle aspiration (FNA) cytology to provide a high level of sensitivity and specificity [2].

During an EUS-guided procedure, an endoscope equipped with an ultrasound (US) transducer and a video camera is inserted and navigated through the gastrointestinal (GI) tract to the stomach and duodenum from which the neighbouring organs such as the pancreas, liver, and biliary ducts can be imaged. However, the small field-of-view, the variability in pancreatobiliary anatomy and the lack of easily definable landmarks make this procedure technically difficult to perform and require skill in both endoscopy and US image interpretation [3].

Multimodal registration of intra-procedure US with pre-procedure images, such as computed tomography (CT) or magnetic resonance (MR) scans, can improve the navigation to specific locations during image-guided procedures by providing additional anatomical context [4, 5]. Initialising the MR/CT to US image registration, for example, by identifying MR- or CT- visible anatomical landmarks on electromagnetically tracked EUS planes [3, 5–7], is critical for robust and accurate registration. However, the relationship between the initialisation-plane selection and registration robustness and accuracy is complex and frequently unintuitive due to: (a) the loss of 3D context in 2D EUS; (b) the limited number of correspondent EUS/CT-visible anatomical point landmarks available with which a registration can be performed; and (c) the high dependency on operator skill and experience [8]. Therefore, pre-procedure simulation to identify anatomical landmarks that are likely to be readily accessible in the EUS field of view and will yield robust registration initialisations may significantly reduce the time and complexity of this step, whilst maximising the accuracy.

Although registration initialisation is common practice in image registration systems, planning tools for optimising multimodal registration accuracy and robustness are not widely available in interventional guidance systems and do not currently exist for EUS-guided procedures. In this paper, we present the first report of a method for simulating the initialisation of a pre-procedure CT- to EUS registration scheme, based on landmarks-to-structure alignment of anatomical landmarks. We identify the optimal position of patient-specific EUS views in terms of enhancing registration accuracy and robustness by ensuring low variation in intra-procedural registration accuracy.

## Methods

### Registration method and initialisation

Landmark-based registration initialisation relies on identifying corresponding landmarks on EUS and CT that represent the upper abdominal anatomy of interest such as organs (e.g. the liver and pancreas); blood vessels (e.g. the splenic artery, splenic vein, and portal vein); and ducts (e.g. the pancreatic duct and the ducts in the biliary tree). Due to the loss of 3D contextual information on EUS, the task of reliably identifying corresponding point landmarks is challenging, subjective, time-consuming and strongly depends on the operator’s experience and skills [8]. Therefore, we propose to align 2D EUS images and 3D pre-procedure CT volumes using a rigid landmark-to-structure registration method, wherein 3D structures (e.g. organ surfaces or vessel/ductal centrelines) are defined on the CT images during the planning stage and, instead of specifying a point-to-point correspondence, the gastroenterologist needs *only identifying the corresponding CT-defined structure for each EUS landmark during the procedure*. The use of landmark-to-structure registration is in practice more feasible as, generally, finding corresponding points requires more time than only defining the structure (i.e. label) to which each point belongs.

**Fig. 1:**
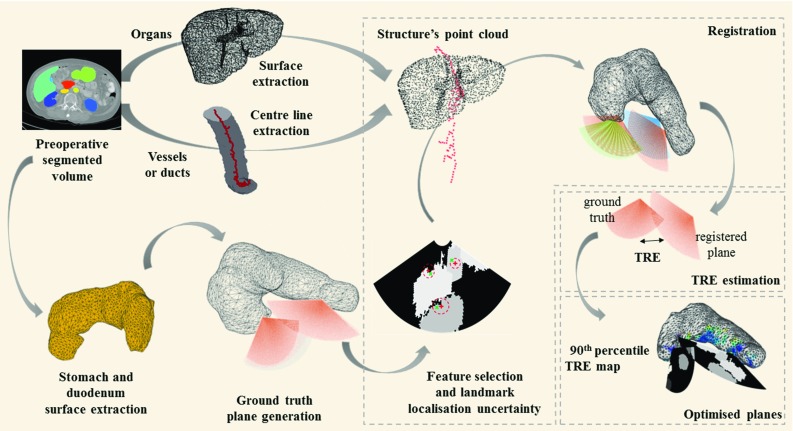
Graphical representation of the planning method (see text for details)

Our approach uses a labelled CT volume where the structures are defined prior to the procedure. In theory, this can be done manually or automatically [9]. Sophisticated semi-automatic tools exist to accelerate manual image segmentation for some applications, but manual segmentation is still impractical for many clinical applications. For the purposes of this study, we assume that a segmented CT (or MRI) volume is available where relevant structures are labelled, without placing any restrictions on how these data are generated. Using this labelled volume, we followed two different strategies depending on the type of structure: (1) for organs, we extracted the surface from the CT labelled volume (see Sect. 3.2 for implementation details); (2) in the case of vessels and ducts, given that the centre of the structure can be easily identified in a 2D US image, we extracted the centreline using a parallel medial-axis thinning method [10]. Both types of structures were represented as a point cloud as illustrated in Fig. 1.

During an EUS-guided procedure, a minimum of three anatomical landmarks need to be manually identified by the gastroenterologist in the EUS images by defining points (i.e. clicking on the screen) and assigned to the corresponding CT/MR structure. The registration method starts with a stochastic initialisation assigning to each US landmark a random point from the corresponding structure’s point cloud and then iteratively refines the point correspondence. In each iteration of refinement, as in the well-known iterative closest point (ICP) algorithm, the method finds an overall rigid transformation that minimises the root-mean-square (RMS) distance between all EUS landmarks and their corresponding point clouds.

### Target registration error estimation

The accuracy of navigation depends on the registration initialisation, and thus, relies on the selection of anatomical landmarks and the uncertainty on landmark localisation [11]. To evaluate plausible EUS plane positions and orientations in 3D (determined by the pose of the transducer), and plausible sets of visible anatomical landmarks, we aim to estimate the corresponding *target registration error* (TRE). Although analytical approximations have been presented to estimate the TRE in point-to-point or surface registration [11, 12], the use of centreline representations preclude their use. Therefore, we estimated the TRE via a MC simulation of landmark localisation error. Figure 1 shows a graphical representation of the proposed method.

Our simulations are constrained to plausible EUS positions. Considering that the endoscope must be in contact with the stomach wall or duodenum to ensure acoustic coupling and obtain a good-quality US image during an EUS-guided procedure, only positions on the surface of these two organs should be considered candidates for registration. For this reason, we used the vertices of the surfaces to sample possible transducer positions during the procedure. From each vertex, considering rotation about the three orthogonal axes, sector view planes were sampled uniformly to simulate views obtainable with an EUS transducer. These planes were considered to be the ground truth for the purposes of the experiments described in Sect. 3.

For each of the EUS view planes, the MC simulations of landmark localisation error enable the estimation of a TRE. Landmarks were automatically extracted depending on the topology of the structure in question (i.e. a vessel/duct or organ). We modelled the uncertainty in landmark localisation as an independent and isotropic Gaussian error with zero mean. Only planes on which three or more features were present were considered in the simulation to ensure that a six-degree-of-freedom rigid transformation could be determined. Simulations were performed using the registration method described in Sect. 2.1. The simulation was repeated to estimate the distribution of TREs from all orientations that contain the same set of features (see Sect. 3 for details).

For each simulation, we calculated two metrics: the TRE between two planes and the TRE for a region of interest. The TRE between two planes (i.e. ground truth and registered plane) was quantified as the RMS error of the Euclidean distances between each corresponding point on the planes (i.e. between corresponding pixels) as follows: $\text {TRE}_\mathrm{plane} = \sqrt{\frac{1}{n}\sum _{i=1}^{n} d({ gt}_{i},{rp}_{i})^2}$, where *n* corresponds to the number of points of the planes and $d({gt}_{i},{rp}_{i})$ to the 3D Euclidean distance between the *i*th point of the ground truth plane *gt* and the *i*th point of the registered plane *rp*. This provided an estimate of the accuracy of registration for a specific plane. The TRE for a region of interest which may lay outside the EUS plane but may be of clinical interest (i.e. lesion in the pancreas) was quantified as the RMS error of the Euclidean distances between each corresponding point on the surfaces of the structures as follows: $\text {TRE}_\mathrm{surface} = \sqrt{\frac{1}{m}\sum _{i=1}^{m} d(s_{i},\mathrm{rs}_{i})^2}$, where *m* corresponds to the number of vertices of the surface and $d(s_{i},\mathrm{rs}_{i})$ to the 3D Euclidean distance between the *i*th vertex of the surface of interest *s* and the *i*th vertex of the registered surface of interest rs. This second measure gives a better estimate of the possible accuracy targeting a region of interest.

### Optimal EUS plane selection

To assist the gastroenterologist during planning of the procedure, we aim to determine the optimal planes likely to produce a robust registration initialisation with a minimised TRE. The mean and variance of TREs obtained from different simulations are both relevant measures to assess the registration initialisation accuracy as a small estimated TRE does not guarantee a robust initialisation with small variance. Therefore, as an alternative, we propose to minimise the 90th *percentile of TREs* instead of the estimated mean TRE. The 90th percentile can be interpreted as the estimated upper bound of the nonparametric 90% prediction interval, such that the optimised EUS plane will yield a 90% probability of achieving the 90th percentile TRE or lower [13]. We believe that this is a more clinically informative measure for procedure planning.

Furthermore, registration error depends on the landmark localisation, which is difficult to generalise to other locations, especially locations distant to the plane, if only one EUS plane is used to initialise registration. Therefore, we investigated whether the addition of a second plane may provide a more robust registration, by performing a second MC simulation. For computational efficiency, pairs of EUS planes were sampled using a Latin hypercube sampling scheme [14], drawing from all possible pairs of planes with at least three identifiable features in each plane. This sampling scheme ensures that the randomly selected values are uniformly distributed over all possible values. Landmark localisation uncertainty was defined as described earlier in Sect. 2.2. Additionally, in practice, the measured relative position and rotation between planes may be subject to tracking errors. We modelled these as Gaussian errors added only to the 3D position and orientation of the second plane (see Sect. 3.2 for implementation details). Then, we registered the pre-procedure point clouds to the automatically extracted landmarks from each sampled EUS plane, again using the same registration method described in Sect. 2.1 to evaluate the difference in TRE due to using multiple planes. The optimised planes were defined as the combination of planes that yield the smallest 90th percentile of TREs.

The reported mean TREs and 90th percentiles for optimal planes were re-estimated from a set of 1000 independent, case-specific, simulations not used in the optimisation to avoid bias in the TRE estimates. Furthermore, to mitigate inter-subject variability, statistical tests were used to determine whether there was an improvement after adding a second plane. In our statistical analysis, we did not use multiple comparison correction.

## Experiments

### Imaging and post-processing

The evaluation of the method was conducted on nine publicly available, manually segmented CT volumes from the MICCAI 2015 workshop and challenge: *multi-Atlas labelling beyond the cranial vault* [15]. The volumes had variable volume sizes from $512\times 512\times 117$ to $512\times 512\times 198$ voxels, variable pixel sizes from $0.59\times 0.59$ to $0.98\times 0.98$ mm, and variable slice thicknesses from 2.5 to 3 mm. From the labelled volumes, the following anatomical landmarks were available: organs (stomach, pancreas, liver, gallbladder and left kidney) and vessels (aorta, inferior vena cava, portal vein and splenic vein). The duodenum was not available in this dataset.

**Fig. 2:**
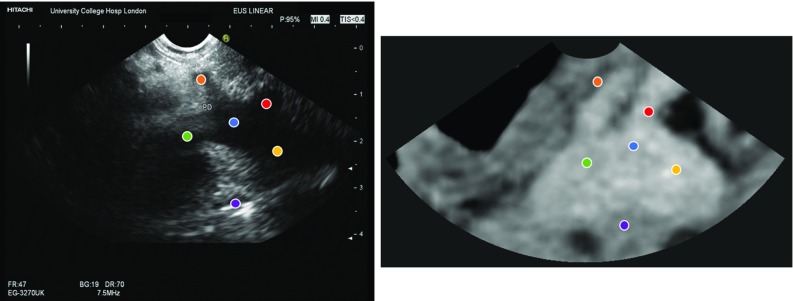
EUS frame and the corresponding CT slice (ground truth) for the first frame of the patient case with the following landmarks: pancreatic duct (orange), bile duct (purple), portal vein (yellow), mesenteric vein (green), splenic vein (red) and confluence of the three veins (blue)

**Fig. 3:**
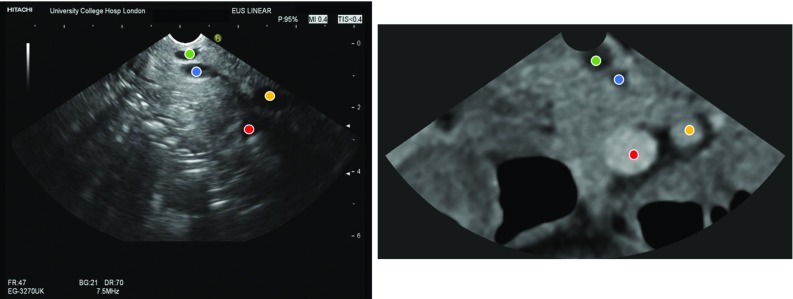
EUS frame and the corresponding CT slice (ground truth) for the second frame of the patient case with the following landmarks: bile duct (green), pancreatic duct (blue), superior mesenteric vein (red) and superior mesenteric artery (yellow)

The feasibility of performing the proposed registration initialisation on clinical data was evaluated retrospectively using data from a 62-year-old female patient who underwent an EUS-guided exploration with FNA. The procedure was performed at University College London Hospital (UCLH) with a Hitachi Preirus EUS console and a Pentax EG-3270UK endoscope with a frequency of 7.5 MHz. During the procedure, approximately 20 min of untracked US data was recorded with a frame resolution of $720\times 576$ pixels and a frame rate of 25 frames per second. Two EUS frames were identified with clear corresponding landmarks localised by two clinical research fellows (LU and GK), confirmed by an experienced consultant gastroenterologist (SP). The region of the stomach (from the pre-procedure CT 3D model) from which the EUS frames were taken was identified by the gastroenterologist and used as a ground truth. In the first frame, using a depth of 4 cm, six landmarks were identified in both CT and US (the pancreatic duct, the splenic vein, the mesenteric vein, the portal vein, and the confluence of the three veins and the common bile duct) with a pixel size of $0.12\times 0.11$ mm (see Fig. 2). In the second frame, four landmarks were identified in both CT and EUS (the pancreatic duct, the bile duct, the superior mesenteric artery and the superior mesenteric vein) with a depth of 6 cm and a pixel size of $0.18\times 0.16$ mm (see Fig. 3). The pre-procedure CT had a volume size of $512\times 512 \times 229$ voxels with a pixel size of $0.55\times 0.55$ mm with a slice thickness of 1 mm. The following labels were extracted automatically using a deep learning approach for multi-organ abdominal segmentation [9] and manually corrected: spleen, right kidney, left kidney, gallbladder, oesophagus, liver, stomach, duodenum and pancreas. Additionally, the following EUS-visible labels were manually segmented: the pancreatic duct, the bile duct, the aorta, the vena cava, the splenic vein, the ampulla, the mesenteric vein and artery, the portal vein and the confluence of portal vein with mesenteric vein and splenic vein and the right adrenal gland.

### Implementation details

To perform the MC simulations, we used a custom-written software implemented in MATLAB (MathWorks, Natick, USA). The isosurfaces of the organs and vessels were extracted from the labelled CT images. To reduce the computational burden, the number of faces on the stomach was reduced such that the average number of vertices was 2638 with an averaged distance between vertices of 5.33 mm, corresponding to approximately half the width of the EUS transducer. The simulated EUS slice sampled from a CT had a view angle of $120^{\circ }$ with a scanning depth of 5 cm, equivalent to standard clinical EUS transducers. For each position, planes within a range of $[{-}\,60, 60]^{\circ }$ with a $20^{\circ }$ step rotation in the three orthogonal axes to the normal of the vertex were considered to simulate physically achievable planes. Therefore, a total of 125 planes were generated for each vertex of the stomach surface and, if available, the duodenum.

The automatically extracted landmarks from vessels corresponded to the feature centroid (i.e. centre of mass) with an added localisation error with a normal distribution of $N(0,\frac{1}{3}(\frac{1}{2} \frac{(w+h)}{2}I)$, where *I* is the identity matrix, and *w* and *h* correspond to the width and height of the bounding box defining the region of the feature visible in the plane, respectively. For organs, the point closest to the mean of all points on the boundary segment visible in the US plane was automatically extracted with an estimated localisation error sampled from a Gaussian distribution $N(0,\sigma I)$, where $\sigma = 2$ mm. Features with cross-sectional areas less than 5 mm$^{2}$ were discarded, as a gastroenterologist would be unable to clearly identify them on EUS images and CT [3]. For each individual plane, 1000 simulations were performed with samples from landmark localisation. For two planes, Gaussian errors with $\sigma = 0.4$ mm and $\sigma = 0.36^{\circ }$ were added to the transducer position and rotation of the second plane, respectively, based on accuracy measurements reported in flexible endoscopes [16]. In this case, twice the number of vertices (stomach surface) were sampled and 100 simulations were performed for each pair of planes.

## Results

Using the nine labelled CT volumes, we performed three comparisons: (a) the result of an optimised plane selection compared to an alternative with a randomly selected plane with at least three landmarks representing a non-optimised approach, (b) the optimised plane compared to planes with the same landmark composition (i.e. the same number of ducts/vessels and same number of organs, as anatomical landmarks) to investigate the dependency of the registration error on the ultrasound image pose as opposed to the landmark composition and (c) the result of using one optimised plane compared to a pair of optimised planes. With a quadratic complexity due to the use of ICP, registration of each plane took an average of 47 ms (using an Intel Xeon CPU E5-1607).

The estimated 90th percentile TREs map of the stomach for each of the nine subjects is illustrated in Table 1. The TRE distributions at the EUS plane and in the pancreas and the 90th percentile TREs are also summarised in Table 1, for the one- and two-EUS-plane registrations. We also include the EUS plane TRE metrics, using the randomly selected planes (i.e. a non-optimised approach), and with the same landmark composition with respect to different poses, in terms of mean and standard deviation (SD).

At a patient level, a Wilcoxon rank-sum test showed a significant difference in median TREs between one and two optimised planes (all *p* values $< 0.03$). Paired student’s *t* tests showed a significant difference between the optimised and non-optimised planes (*p* value $< 0.01$), and between optimised planes and planes with the same landmark composition (*p* value $< 0.01$), as well as a significant difference between one- and two-plane TREs calculated on the pancreas (*p* value $< 0.01$).

Additionally, we modelled the effect of landmark composition on the 90th percentile TRE as a multiple linear regression with two factors: the number of ducts and the number of organs. Under this model, the 90th percentile TRE decreased by 8.47 mm per additional duct landmark and 9.10 mm per organ landmark. The within-group standard deviation of the 90th percentile TRE from different poses after accounting for the number and composition of landmarks was 16 mm. These results suggest that both the landmark composition and the specific pose are important factors to determine the accuracy.

In the second experiment, we assessed the feasibility of the method by analysing the retrospective data taken during the EUS-guided pancreas intervention. The 90th percentile TRE map of the stomach and duodenum of the patient, the mean TREs, 90th percentile TRE and TRE in the pancreas for both simulated and obtained with the real EUS frame are summarised in Table 2. Note that since it is a retrospective study, the optimal US frames were not available. The distribution of TRE and the 90th percentile TRE both had a skewed distribution along right tale (skewness $= 2.38$ and 0.72, respectively). Furthermore, we used the retrospective data to evaluate the correlations between the number and types of landmarks and the 90th percentile TRE using the Pearson’s linear correlation coefficient (CC) considering all vertices. Overall, the number of landmarks was correlated with the 90th percentile TRE with a CC of $-$ 0.71. The number of ducts and vessels was correlated with the 90th percentile TRE with a CC of $-$ 0.68 and the number of organs with a CC of $-$ 0.10.

**Table 1:** Mean and 90th percentile of TREs (± SD) in mm for nine cases with one and two optimised planes (TRE ± SD, 90th p.), the expected mean plane TRE of a non-optimised approach (Non-opt.), the TRE when using the same landmark composition (Comp. TRE) and the expected mean TRE (± SD) on the vertices of the pancreas (Pancreas)

	Case 1	Case 2	Case 3	Case 4	Case 5
90th p. TREs	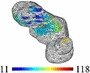	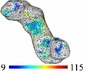	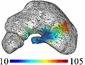	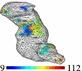	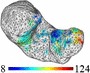
TRE±SD 1p.	$7.52\pm 2.92$	$6.47\pm 2.56$	$8.27\pm 1.86$	$6.30\pm 2.39$	$5.79\pm 1.95$
90th p. 1p.	11.27	8.99	10.50	9.60	8.57
Pancreas 1p.	$16.93\pm 8.57$	$10.88\pm 5.34$	$14.99\pm 9.89$	$18.28\pm 8.36$	$10.61\pm 5.45$
Non-opt. 1p.	$49.75\pm 19.99$	$41.16\pm 17.03$	$46.19\pm 18.95$	$47.63\pm 19.57$	$43.20\pm 16.81$
Comp. TRE	$35.88\pm 11.98$	$27.73\pm 11.26$	$24.43\pm 8.07$	$17.48\pm 4.14$	$7.85\pm 2.21$
Opt. planes	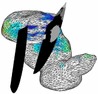	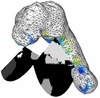	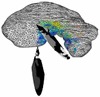	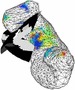	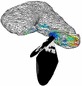
TRE ± SD 2p.	$5.92\pm 2.36$	$4.91\pm 1.96$	$5.41\pm 2.50$	$4.71\pm 1.94$	$5.57\pm 1.39$
90th p. 2p.	8.63	7.26	7.54	7.06	7.37
Pancreas 2p.	$9.31\pm 3.90$	$5.49\pm 2.65$	$5.72\pm 1.84$	$6.22\pm 2.71$	$8.66\pm 1.60$
Non-opt 2p.	$20.76\pm 10.8$	$20.54\pm 14.30$	$24.50\pm 17.09$	$28.39\pm 18.91$	$26.52\pm 15.76$

	Case 6	Case 7	Case 8	Case 9	
90th p. TREs	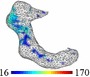	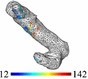	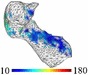	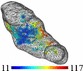	
TRE ± SD 1p.	$10.64\pm 4.61$	$9.49\pm 2.78$	$7.67\pm 2.6$	$9.53\pm 1.91$	
90th p. 1p.	16.95	12.73	10.99	11.54	
Pancreas 1p.	$21.76\pm 10.35$	$16.46\pm 7.87$	$15.35\pm 7.21$	$18.67\pm 4.99$	
Non-opt 1p.	$21.98\pm 24.66$	$38.72\pm 21.20$	$43.72\pm 23.18$	$42.13\pm 15.94$	
Comp. TRE	$15.58\pm 8.34$	$30.36\pm 15.44$	$23.13\pm 10.13$	$10.71\pm 2.82$	
TRE ± SD 2p.	$6.09\pm 1.23$	$5.15\pm 2.49$	$4.58\pm 1.55$	$4.54\pm 1.56$	
90th p. 2p.	7.30	8.93	6.95	6.58	
Pancreas 2p.	$6.62\pm 1.86$	$4.63\pm 1.68$	$5.42\pm 1.81$	$5.98\pm 2.09$	
Non-opt 2p.	$25.45\pm 19.31$	$21.56\pm 16.14$	$17.32\pm 15.67$	$22.32\pm 14.59$	

White regions on the wireframe mesh of the stomach correspond to positions where fewer than three features were identifiable. The 90th percentile TREs map corresponds to the simulation with one single ultrasound plane

**Table 2:** Mean and 90th percentile of TREs (in mm) and the expected TRE at the surface of the pancreas for the two US frames obtained from a patient case

90th percentile TRE map of the stomach	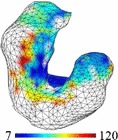	
	Frame 1	Frame 2
Simulated		
TRE ± SD plane	$11.55\pm 3.60$	$23.08\pm 5.09$
90th p. TRE plane	16.76	29.17
TRE ± SD pancreas	$65.76\pm 28.07$	$127.58\pm 34.57$
US frame		
TRE ± SD plane	$8.87\pm 1.82$	$13.27\pm 5.76$
90th p. TRE plane	11.03	21.60
TRE ± SD pancreas	$65.77\pm 12.36$	$62.28\pm 42.91$

## Discussion

This work proposes a planning tool for image-guided EUS therapy to the pancreas. We propose to (1) use a landmarks-to-structure rigid registration method to relax the dependency on landmark selection and localisation; (2) predict the TRE on the surface of the stomach and the duodenum (if available) via MC simulations; and (3) use the 90th percentile of TREs to determine optimal EUS frames for a robust and accurate registration.

Results suggest that optimised planes can potentially provide a more robust registration in terms of TRE compared to a non-optimised approach and compared to planes with the same landmark composition but a different pose. Additionally, using two planes can also provide a better registration accuracy in regions of interest such as the pancreas. The estimated accuracy of registration can also inform the gastroenterologist’s decision whether one or two EUS initialisation planes should be used, where these planes should be located and what TRE can be expected with high confidence.

The evaluation in a clinical setting was performed using retrospective data, where the optimal plane was not available. The purpose of this experiment was to demonstrate the feasibility and application of the method in a real clinical scenario. Although with the limited data available, a rigorous validation of the TRE estimation was not possible, results showed differences between the predicted plane TRE and the calculated TRE. Based on our observations, we suspect this was in part due to the number of landmarks that were automatically identified from the CT, which were not visible on EUS images (Fig. 2), and may also be attributed to our conservative overestimate of landmark uncertainty for segmentation-defined landmarks, which was higher than the manual variation (Fig. 3). Both issues could be solved using more accurate models of the landmark distributions on the sector-plane view and are interesting subjects of future investigation.

Our sector-view-plane simulation approach could be applied in a variety of EUS-landmark-based registrations. In this work, we registered the US frames to the pre-procedural CT using an ICP method, as it is widely known and used for multimodal registration initialisation [5, 8]. Other algorithms, such as robust ICP [17, 18], have a similar unintuitive relationship between plane selection and TRE and therefore could also benefit from these simulations.

Abdominal CT organ segmentation uncertainty could affect simulation results in multiple ways. First, different segmentation errors could yield different optimal planes and TREs. This is expected and not a problem because the same segmentation is used for planning and intra-procedural guidance, and different segmentations may, in fact, require different initialisation planes. Second, segmentation uncertainty can increase the effective landmark localisation uncertainty. Our simulations include a model of the distribution of the EUS-localised landmarks $l_{i}$ on the simulated sector views, represented as a distribution of offsets $o_{i} = l_{i}-s_{i}$ from segmentation-defined points, $s_{i}$. In Sect. 2.2, we modelled the EUS landmark localisation distribution relative to EUS-defined ground truth points, $e_{i}$. If segmentation uncertainty induces variability in the segmentation-defined points, $s_{i}$, then the distribution of offsets between EUS-defined and segmentation-defined points [i.e. $o_{i} = l_{i}-s_{i} = (l_{i}-e_{i})+(e_{i}-s_{i})$] is the sum of the two distributions, which in general will have a larger magnitude and could be modelled in future simulations. On the other hand, considering organ deformation requires a robust non-rigid registration method, which, to the best of our knowledge, has not been proposed yet for the application of interest. This could also reduce the potential limitation such as availability of landmarks during the procedure caused by out-of-plane rotation. The robustness of the planes could be further evaluated by assessing different distributions for modelling the uncertainties. However, for the purposes of this study, some simplifications such as using Gaussian distributions with zero mean, were needed to show and demonstrate the feasibility of the method.

In this proof-of-concept work, we used the TRE between the registered and ground truth plane as a measure of registration accuracy for optimisation. Regions of clinical interest, such as a target lesion or an anatomical structure that lies far from the optimised plane, may of course be registered with a different TRE (e.g. pancreas in Table 1) and compromise the accuracy of the navigation in that area. In this case, the mean pancreas TRE was typically larger than the mean EUS-plane TRE, by 95% for the one-plane registration and by only 23% for the two-plane registrations. When specific clinical targets are defined before the procedure, the methodology described could be easily adopted to directly minimise the TRE on the clinical target (estimated to be useful with an accuracy within 5 cm) yielding a patient-and-procedure-specific plan.

## Conclusion

This work proposes a planning method for endoscopic procedures involving the gastrointestinal tract with the aim to improve the navigation accuracy during EUS procedures. In conclusion, results show that optimised planes provide a more robust initialisation and that there is a time–accuracy trade-off in opting to use one or two planes for registration initialisation. We evaluated the use of the method in a clinical setting retrospectively using US images from a EUS case.

Future work will include more patient data to further assess the proposed method in a clinical environment for EUS-guided procedures.

## Electronic supplementary material

Below is the link to the electronic supplementary material.

Supplementary material 1 (avi 44799 KB)

Supplementary material 2 (avi 113005 KB)

Supplementary material 3 (avi 113245 KB)

Supplementary material 4 (avi 113175 KB)
